# Intratracheal transplantation of human umbilical cord blood-derived mesenchymal stem cells attenuates *Escherichia coli*-induced acute lung injury in mice

**DOI:** 10.1186/1465-9921-12-108

**Published:** 2011-08-15

**Authors:** Eun Sun Kim, Yun Sil Chang, Soo Jin Choi, Jin Kyu Kim, Hey Soo Yoo, So Yoon Ahn, Dong Kyung Sung, Soo Yoon Kim, Ye Rim Park, Won Soon Park

**Affiliations:** 1 Department of Pediatrics, Samsung Medical Center, Sungkyunkwan University School of Medicine, Seoul, Korea; 2 Samsung Biomedical Research Institute, Samsung Medical Center, Seoul, Korea; 3 Biomedical Research Institute, MEDIPOST Co., Ltd., Seoul, Korea

**Keywords:** Acute respiratory distress syndrome, Infection, Inflammation, Escherichia coli, Animal

## Abstract

**Background:**

Human umbilical cord blood (UCB)-derived mesenchymal stem cells (MSCs) attenuate hyperoxic neonatal lung injury primarily through anti-inflammatory effects. We hypothesized that intratracheal transplantation of human UCB-derived MSCs could attenuate *Escherichia coli (E. coli)*-induced acute lung injury (ALI) in mice by suppressing the inflammatory response.

**Methods:**

Eight-week-old male ICR mice were randomized to control or ALI groups. ALI was induced by intratracheal *E. coli *instillation. Three-hours after *E. coli *instillation, MSCs, fibroblasts or phosphate-buffered saline were intratracheally administered randomly and survival was analyzed for 7 days post-injury. Lung histology including injury scores, myeloperoxidase (MPO) activity, and protein levels of interleukin (IL)-1α, IL-1β, IL-6, tumor necrosis factor (TNF)-α, and macrophage inflammatory protein (MIP)-2 as well as the wet-dry lung ratio and bacterial counts from blood and bronchoalveolar lavage (BAL) were evaluated at 1, 3, and 7 days post-injury. Levels of inflammatory cytokines in the lung were also profiled using protein macroarrays at day 3 post-injury which showed peak inflammation.

**Results:**

MSC transplantation increased survival and attenuated lung injuries in ALI mice, as evidenced by decreased injury scores on day 3 post-injury and reduced lung inflammation including increased MPO activity and protein levels of IL-1α, IL-1β, IL-6, TNF-α, and MIP-2 on day 3 and 7 post-injury. Inflammatory cytokine profiles in the lungs at day 3 post-injury were attenuated by MSC transplantation. MSCs also reduced the elevated lung water content at day 3 post-injury and bacterial counts in blood and BAL on day 7 post-injury.

**Conclusions:**

Intratracheal transplantation of UCB-derived MSCs attenuates *E. coli*-induced ALI primarily by down-modulating the inflammatory process and enhancing bacterial clearance.

## Background

Acute respiratory distress syndrome (ARDS), a severe form of acute lung injury (ALI), is an acute respiratory failure in critically ill patients [1]. The mortality of ARDS/ALI remains unacceptably high because of the lack of effective treatments [2]. Infection is the most common cause of ARDS/ALI and results in a higher mortality rate than noninfectious ALI [3,4]. The inflammatory process plays a key role in the pathogenesis of both infectious and noninfectious ALI, and the degree of acute inflammation is strongly correlated with outcome [5].

Recently, transplantation of various stem or progenitor cells such as bone marrow (BM)-derived mesenchymal stem cells (MSCs) or endothelial progenitor cells was reported to reduce mortality and attenuate ALI induced by endotoxins or sepsis in a rodent model [6-8]. This attenuation was associated with moderation of the inflammatory reactions that accompany ALI, with variable anti-bacterial effects [9,10]. These studies indicate that MSC treatment could be a new therapeutic modality for the treatment of ALI. Among the various sources of stem cells, human umbilical cord blood (UCB) provides readily available MSCs with low immunogenicity [11-13]. Therefore, human UCB-derived MSCs are regarded as a viable candidate source for cell therapy; however, this has yet to be studied in an *in vivo *ARDS/ALI model. We previously demonstrated that intratracheal transplantation of human umbilical cord blood (UCB)-derived MSCs attenuates hyperoxic lung injury in newborn rats through anti-inflammatory effects rather than direct regeneration [14,15].

We thus hypothesized that intratracheal transplantation of human UCB-derived MSCs could attenuate *Escherichia coli (E. coli)*-induced ALI in adult mice, and if so, the protective mechanism might be primarily mediated by anti-inflammatory effects. In this study, we used a clinically relevant mouse model of infectious ARDS/ALI with Gram-negative bacterial pneumonia and sepsis induced by intratracheal instillation of *E. coli *[16]. We examined the effects of intratracheal delivery of human UCB-derived MSCs on survival, histology, and lung inflammation in mice with *E. coli*-induced ALI. Histological injury scores and myeloperoxidase (MPO) activity in lung homogenates were evaluated. Interleukin (IL)-1α, IL-1β, IL-6, tumor necrosis factor (TNF)-α, and macrophage inflammatory protein (MIP)-2 protein levels in lung homogenates were measured serially by ELISA. Mouse lungs showed peak inflammation three days after injury, at which time they were profiled using protein macroarray analysis. We chose this high-output proteomics approach for identifying proteins of interest in an effort to elucidate the potential mechanisms by which human UCB-derived MSCs modulate inflammatory responses. The wet-dry lung ratio and bacterial concentrations in bronchoalveolar lavage (BAL) and blood specimens after UCB-derived MSC transplantation were also examined.

## Methods

### Cell preparation

This study was approved by the Institutional Review Board of Samsung Medical Center and by Medipost, Co., Ltd, Seoul, Korea. UCB is the most promising source of MSCs because of its easy availability and the low immunogenicity of the cells; MSCs from UCB can be administered between HLA-incompatible individuals due to these cells' immune-modulatory properties, without alloreactive lymphocyte proliferative responses [11-13]. In the present study, MSCs were isolated and cultivated from human UCB as previously reported [14,15]. UCB was collected from umbilical veins after neonatal delivery with informed consent from pregnant mothers. The cells were shown to express CD105 and CD73 (99.6% and 96.3%, respectively) but not CD34, CD45, or CD14 (0.1%, 0.2%, and 0.1%, respectively). The cells were positive for HLA-AB (96.8%) but not HLA-DR (0.1%). Human UCB-derived MSCs differentiated into various cell types such as respiratory epithelium, osteoblasts, chondrocytes and adipocytes upon specific *in vitro *induction stimuli [15]. We confirmed the differentiation potential and karyotypic stability of the human UCB-derived MSCs up to the 11^th ^passage [15]. In this study, 5^th ^passage human UCB-derived MSCs from a single donor were used for the transplantions.

Human fibroblasts (MRC-5; Korean Cell Line Bank No.10171) were obtained from the Korean Cell Line Bank (Seoul, Korea) and cultured in α-MEM medium supplemented with 10% fetal bovine serum.

### Bacterial preparation

We used *E. coli *as the source of infection because it is a common cause of gram-negative bacterial lung infection [16]. The *E. coli *strain E69 was generated by Pl transduction of the *E. coli *K12 outer membrane protein A (Omp A) gene into an Omp A^- ^mutant of RS 218, isolated from the CSF of a newborn with *E. coli *meningitis (a gift from Dr. Kwang Sik Kim, Johns Hopkins Hospital, Baltimore, MD, USA) [17]. *E. coli *was cultured overnight in 10 mL of brain heart infusion broth (BHI, Difco Laboratories, Detroit, MI, USA) at 37°C. The bacteria were then diluted in BHI media and grown for 1 h to mid-logarithmic phase. The suspension was centrifuged for 10 min at 5,000 g and washed in phosphate-buffered saline (PBS). Optical density was measured, and the bacteria samples were adjusted to the desired concentration. The final *E. coli *preparation contained 10^7 ^colony forming units (CFUs) in 0.05 mL PBS [18].

### Animal model

All of the experimental protocols were approved by the Institutional Animal Care and Use Committee of Samsung Biomedical Research Institute. The study followed the institutional and National Institutes of Health guidelines for laboratory animal care.

Eight-week-old male ICR mice were purchased from Orient Co. (Seoul, Korea) and housed in individual cages with free access to water and laboratory chow. Animals were divided into four groups: sham control (S, n = 57), *E. coli*-induced ALI control (E, n = 125), ALI with fibroblast transplantation (F, n = 58), and ALI with human UCB-derived MSCs transplantation (M, n = 104).

To induce ALI, mice were anesthetized with an intraperitoneal injection of a mixture of ketamine and xylazine (45 mg/kg and 8 mg/kg, respectively). Briefly, each mouse was restrained at a 70° angle against a plastic wall, an otoscope was employed to visualize the vocal cords, and intubation was performed with a 20-gauge central catheter (Leader Cath 20 G × 8 cm; Vygon, Paris, France). *E. coli *at 10^7 ^CFUs in 0.05 mL PBS was administered, followed by 2 cm H_2_O-pressure air inflation to ensure an even bacterial distribution. After the *E. coli *instillation procedure, which was completed within 30 sec for each mouse, the catheters were removed and the animals were allowed to recover and subsequently returned to their dams.

For cell transplantation, 1 × 10^5 ^cells (MSCs in M or fibroblasts in F) in 0.05 mL PBS were administered intratracheally three hours after the *E. coli *instillation. Mice in groups S and E received the same volume of PBS intratracheally. For intratracheal transplantation, the animals were anesthetized and the catheter was placed as described above. After intratracheal transplantation, the catheter was removed and the mice were allowed to recover and subsequently returned to their dams.

Intraperitoneal antibiotics (ceftriaxone, 100 mg/kg once a day) were administered for three consecutive days after the injury. Mice were sacrificed at post-injury days 1, 3 and 7, and each animal was allocated to a morphometric or biochemical group. Survival rates were assessed for seven days after injury in the 7-day experimental groups.

### Tissue preparation

Lung tissue was prepared from surviving animals at post-injury days 1, 3 and 7. The mice were anesthetized with sodium pentobarbital (100 mg/kg), and the lungs and heart were exposed via thoracotomy, followed by transcardiac perfusion with ice-cold PBS.

The lungs were fixed by tracheal instillation of 4% formaldehyde with a constant inflation pressure of 20 cm H_2_O. The trachea was ligated, and the lungs were removed and immersed in 4% formaldehyde overnight at room temperature. Both lungs were embedded in paraffin, and transverse serial sections (4 μm thick) were prepared for morphometric analyses. For the biochemical analyses, the lungs were excised, frozen in liquid nitrogen, and homogenized.

### Morphometric analyses

Four-micrometer-thick sections were stained with hematoxylin and eosin. Two sections per mouse were randomly chosen for the analysis, and three random microscopic fields of the distal lung were evaluated by a blinded observer. Lung injury was scored according to the following four categories: alveolar congestion, hemorrhage, neutrophil infiltration into the airspace or vessel wall, and alveolar wall thickness/hyaline membrane formation. Each category was graded on a five point scale: 0 = minimal injury, 1 = injury up to 25% of the field, 2 = injury up to 50% of the field, 3 = injury up to 75% of the field, and 4 = diffuse injury [19].

### Myeloperoxidase (MPO) activity assay

MPO activity in the homogenized lung tissues was measured as described by Gray et al [20]. One unit of MPO activity was defined as the quantity of enzyme needed to degrade 1 μmol of peroxide/min at 25°C.

### Enzyme-linked immunosorbent assay (ELISA)

The frozen lungs were homogenized in cold buffer (50 mM Tris-HCl, pH 7.4) with 1 mM EDTA, 1 mL EGTA, 1 mM PMSF, 42 mM KCl, and 5 mM MgCl_2_. The samples were centrifuged at 8,000 g for 20 min at 4°C to remove cellular debris. The protein content in the supernatant was measured using the Bradford method with a bovine serum albumin (Sigma-Aldrich, St. Louis, MO, USA) standard. Lung interleukin (IL)-1α, IL-1β, IL-6, tumor necrosis factor (TNF)-α, and macrophage inflammatory protein (MIP)-2 levels were measured using the Milliplex MAP ELISA Kit according to the manufacturer's protocol (Millipore, Billerica, MA, USA).

### Protein macroarray

Each lung lysate was analyzed using a mouse cytokine array kit (Proteome Profiler™; R&D Systems, Minneapolis, MN, USA). A total of 250 μg of lysate was incubated in the nitrocellulose membrane array overnight at 4°C. After washing away the unbound protein, the array was incubated with a cocktail of phospho-site-specific biotinylated antibodies for 2 h at room temperature, followed by streptavidin-HRP for 30 min. Signals were visualized with chemiluminescent reagents (Amersham Biosciences, Pittsburgh, PA, USA), and recorded on X-ray film. The arrays were scanned, and optical densities were measured using Image J software (NIH) and compared among the experimental groups.

The protein macroarray analysis included inflammatory cytokines of interest, including complement 5a (C5a), the soluble form of intercellular adhesion molecule (sICAM)-1, IL-1α, IL-1β, IL-6, IL-16, interferon-inducible protein (IP)-10, the murine analogue of monocyte chemoattractant protein (MCP)-1 (JE), MCP-5, MIP-1α, MIP-1β, MIP-2, regulated upon activation normal T-expressed and presumably secreted (RANTES), tissue inhibitor of metalloproteinase (TIMP)-1, TNF-α, and triggering receptor expressed on myeloid cells (TREM)-1.

### Wet-dry lung ratios

The lungs from each animal were removed, placed into a microtube, and weighed. The lungs were then dried at 60°C for 72 h and weighed again. The wet lung mass divided by the dry lung mass represented the wet-dry lung ratio, which indicates the fraction of wet lung weight due to water.

### Bacterial quantification

Bacterial concentrations were measured in BAL fluid and blood from the E, F, and M groups at post-injury days 1, 3 and 7. Mice were anesthetized, thoracotomy was performed as described above, and intracardiac blood was obtained aseptically, followed by transcardiac perfusion with PBS. BAL fluids were obtained via an aseptic saline irrigation.

Bacteria CFU levels in BAL fluids and blood were measured at dilutions of 10^-3^~10^-6 ^plated on BHI agar after overnight incubation at 37°C.

Assessment of direct inhibition of bacterial growth by MSCs or fibroblast was done by counting CFUs *in vitro*. Dulbecco's modified Eagle's medium (DMEM) was used as a control medium and human fibroblasts (MRC-5; Korean Cell Line Bank No.10171) were used as a control cells. In brief, each 3 well-plates of DMEM, fibroblast, and MSCs in 12-well plates (10^5 ^cells per well) in BHI media were infected with 10^3 ^CFUs *E. coli *and incubated for 6 hours in humidified CO_2 _incubator, then aliquots of culture medium were taken from each well, serially diluted with sterile PBS, and plated on agar plates. Colonies were counted after overnight incubation at 37°C.

### Statistical analyses

The data are expressed as the mean ± SEM. Survival rates were compared using the Kaplan-Meier analysis followed by a log rank test. For continuous variables with a normal distribution, the groups were compared using a *t*-test with a Bonferroni correction. Continuous variables that were not normally distributed were analyzed using the Wilcoxon rank test with a Bonferroni correction. All data were analyzed using SPSS version 12.0 (SPSS Inc., Chicago, IL, USA). Values of *p *< 0.05 were considered statistically significant.

## Results

### Survival rate

**Figure 1:**
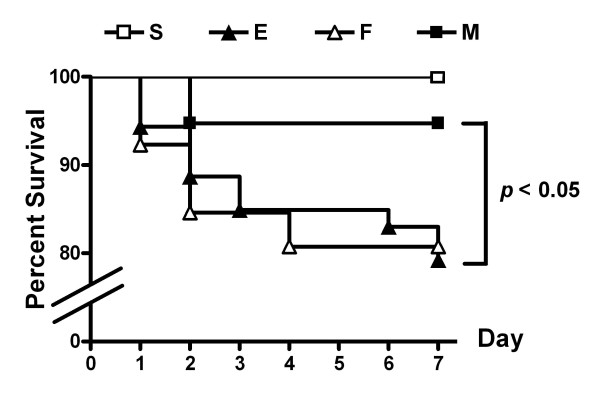
**Survival Rates**. Survival rates determined using the Kaplan-Meier analysis followed by a log rank test. S, sham group; E, *E. coli*-induced ALI control group; F, fibroblast transplant ALI group; M, human UCB-derived MSC transplant ALI group.

Despite antibiotic treatment, *E. coli*-induced ALI (E) significantly reduced the survival rate (79%, 42/53 mice surviving) at post-injury day 7 compared to the 100% survival rate (22/22) of the sham control group (S). The reduced survival rate observed in E (*p *< 0.05 vs. S) improved with MSC treatment (M) (95%, 36/38 mice surviving; *p *> 0.05 vs. S and *p *< 0.05 vs. E), but not with fibroblast treatment (F) (*p *< 0.05 vs. S, *p *= 0.91 vs. E) (Figure 1).

### Lung histology, injury scores, and MPO activity

**Figure 2:**
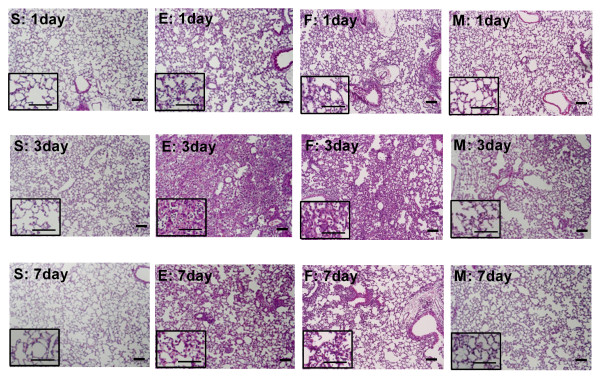
**Gross Histologic Evaluation**. Photomicrographs of hematoxylin and eosin staining of each group at three time points. (Scale bar = 25 μm and 10 μm in the magnified photograph).

**Figure 3:**
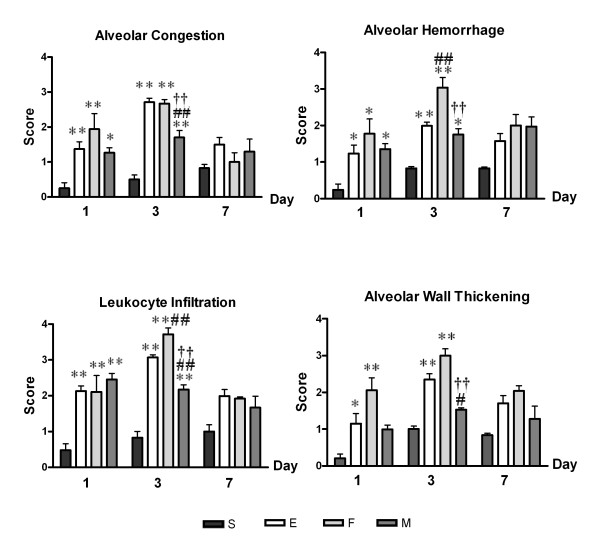
**Lung Injury Scores**. Lung injury scores according to each category. Data: mean ± SEM, n = 6, 10, 4 and 10 in S, E, F, and M groups at day 1, respectively; n = 5, 9, 4 and 8 in S, E, F, and M groups at day 3, respectively; n = 7, 9, 4 and 8 in S, E, F, and M groups at day 7, respectively; ** *p *< 0.01 vs. S group; * *p *< 0.05 vs. S group; ## *p *< 0.01 vs. E group; # *p *< 0.05 vs. E group; †† *p *< 0.01 vs. F group; † *p *< 0.05 vs. F group.

Representative photomicrographic lung histology in group E showed increased congestion and cellular infiltration at post-injury days 1, 3 and 7, with peak inflammatory activity observed at day 3 compared with that of S (Figure 2). To quantify the differences, lung injury was scored according to the degree of alveolar congestion, hemorrhage, neutrophil infiltration, and wall thickening (Figure 3).

Group E showed significant increases in all injury scores at post-injury day 1 (*p *< 0.01 vs. S for alveolar congestion and leukocyte infiltration; *p *< 0.05 vs. S for alveolar hemorrhage and wall thickening) and day 3 (*p *< 0.01 vs. S for all scores) but not at day 7 (*p *> 0.05 vs. S in all scores). MSC transplantation (M) significantly attenuated the ALI-induced increases in injury scores at post-injury day 3 (*p *< 0.01 vs. E for alveolar congestion and leukocyte infiltration; *p *< 0.05 vs. E for alveolar wall thickening), except for alveolar hemorrhage (*p *> 0.05 vs. E), while fibroblast transplantation (F) did not attenuate and even aggravated the injury scores for alveolar hemorrhage and leukocyte infiltration (*p *< 0.01 vs. E) at post-injury day 3.

**Figure 4:**
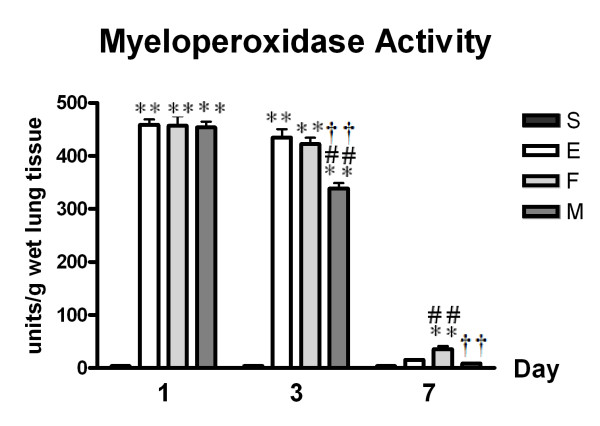
**Lung Myeloperoxidase Activity**. Data: mean ± SEM, n = 4, 4, 4 and 4 in S, E, F, and M groups at day 1, respectively; n = 4, 4, 4 and 4 in S, E, F, and M groups at day 3, respectively; n = 5, 5, 5 and 5 in S, E, F, and M groups at day 7, respectively; ** *p *< 0.01 vs. S group; ## *p *< 0.01 vs. E group; †† *p *< 0.01 vs. F group.

MSC transplantation (M, *p *< 0.01 vs. E), but not fibroblast transplantation (F, *p *> 0.05 vs. E), significantly attenuated ALI-induced increases in lung MPO activity (an indication of neutrophil accumulation) (E, *p *< 0.01 vs. S) at post-injury day 3 (Figure 4).

### Proinflammatory cytokines

**Figure 5:**
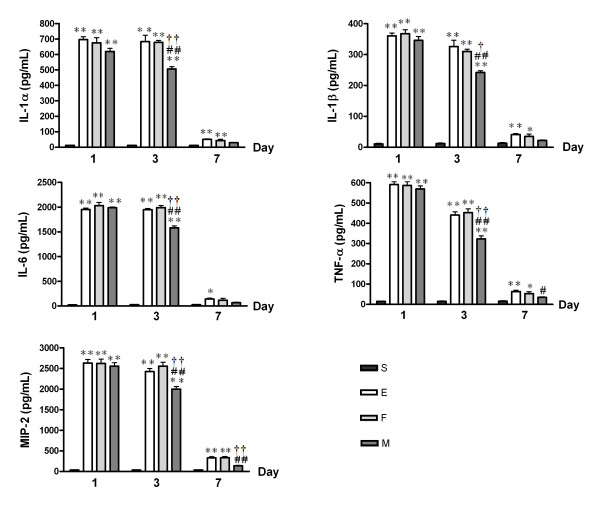
**Lung Inflammatory Cytokine Protein Levels According to ELISA**. Histograms for IL-1α, IL-1β, IL-6, TNF-α, and MIP-2 proteins in the lungs. Data: mean ± SEM, n = 4, 4, 4 and 4 in S, E, F, and M groups at day 1, respectively; n = 4, 4, 4 and 4 in S, E, F, and M groups at day 3, respectively; n = 5, 5, 5 and 5 in S, E, F, and M groups at day 7, respectively; ** *p *< 0.01 vs. S group; * *p *< 0.05 vs. S group; ## *p *< 0.01 vs. E group; # *p *< 0.05 vs. E group; †† *p *< 0.01 vs. F group; † *p *< 0.05 vs. F group.

The protein levels of IL-1α, IL-1β, IL-6, TNF-α, and MIP-2 measured by ELISA in lung homogenates were significantly higher in group E compared to S at post-injury days 1 and 3 (E, *p *< 0.01 vs. S for all), and to a lesser extent at post-injury day 7 (E, *p *< 0.01 vs. S for IL-1α, IL-1β, TNF-α, and MIP-2; *p *< 0.01 vs. S for IL-6). This ALI-induced increase in cytokine levels was significantly attenuated by MSC transplantation (M) but not by fibroblast transplantation (F) at post-injury days 3 (M, *p *< 0.01 vs. E), and 7 (M, *p *> 0.05 vs. S) (Figure 5).

### Lung cytokine profiles

**Figure 6:**
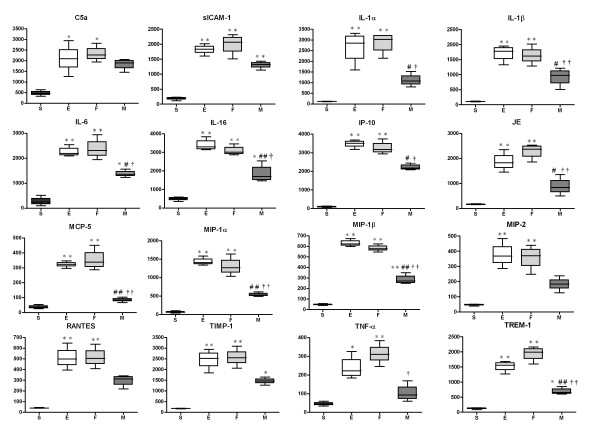
**Protein Macroarray Data for Each Group at Day 3**. Densitometric histograms for C5a, sICAM-1, IL-1α, IL-1β, IL-6, IL-16, IP-10, JE, MCP-5, MIP-1α, MIP-1ß, MIP-2, RANTES, TIMP-1, TNF-α, and TREM-1. Data: mean ± SEM, n = 4, 4, 4 and 4 in S, E, F, and M groups, respectively; ** *p *< 0.01 vs. S group; * *p *< 0.05 vs. S group; ## *p *< 0.01 vs. E group; # *p *< 0.05 vs. E group; †† *p *< 0.01 vs. F group; † *p *< 0.05 vs. F group.

Lung homogenates at post-injury day 3 were used for a mouse macroarray experiment to profile various inflammatory cytokine proteins involved in the peak inflammatory reactions induced by ALI with *E. coli *instillation. Protein levels of inflammatory cytokines from lung homogenates, including C5a, sICAM-1, IL-1α, IL-1β, IL-6, IL-16, IP-10, JE, MCP-5, MIP-1α, MIP-1β, MIP-2, RANTES, TIMP-1, TNF-α, and TREM-1, were significantly elevated in E (*p *< 0.01 vs. S, except for TNF-α: *p *< 0.05 vs. S). MSC transplantation reduced the levels of C5a, MIP-2, RANTES, and TNF-α to S levels (M, *p *> 0.05 vs. S), markedly reduced the levels of IL-16, MCP-5, MIP-1α, MIP-1β, and TREM-1 (M, *p *< 0.01 vs. E), and significantly reduced the levels of IL-1α, IL-1β, IL-6, IP-10, and JE (*p *< 0.05 vs. E). Fibroblast transplantation did not attenuate this increase for any cytokine (F, *p *< 0.01 vs. S; *p *> 0.05 vs. E) (Figure 6).

### Lung water content

**Figure 7:**
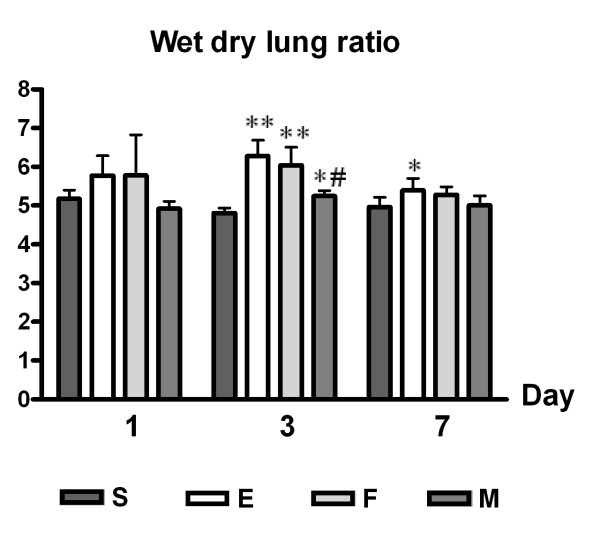
**Lung Water Content (Wet-Dry Lung Ratio)**. Lung water content (wet-dry lung ratio) in mice treated with human UCB-derived MSCs, human fibroblasts, or PBS after ALI induction by intratracheal *E. coli *instillation. Data: mean ± SEM, n = 7, 14, 6 and 15 in S, E, F, and M groups at day 1, respectively; n = 5, 17, 5 and 18 in S, E, F, and M groups at day 3, respectively; n = 10, 15, 12 and 13 in S, E, F, and M groups at day 7, respectively; ** *p *< 0.01 vs. S group; * *p *< 0.05 vs. S group; # *p *< 0.05 vs. E group.

*E. coli*-induced ALI (E) increased lung water content expressed as the wet-dry lung ratio, which suggested increased permeability compared S at post-injury. The increase in lung water content was marked at day 3 (E, *p *< 0.01 vs. S) and to a lesser extent at day 7 (E, *p *< 0.05 vs. S). MSCs transplantation (M, *p *< 0.05 vs. E) but not fibroblast transplantation (F, *p *> 0.05 vs. E) reduced lung water content significantly at post-injury day 3 (Figure 7).

### Bacterial counts

**Figure 8:**
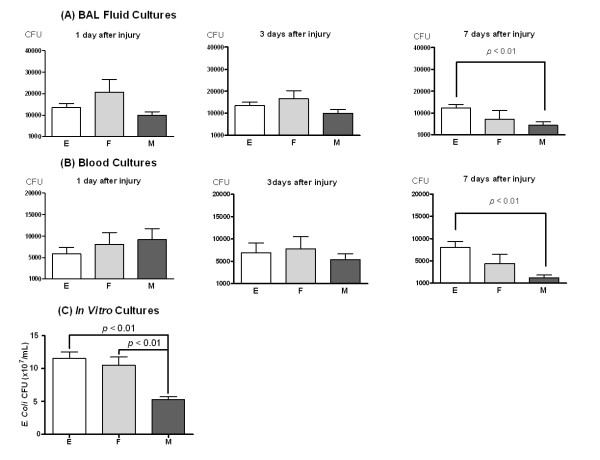
**Bacterial Growth *in vivo *(A and B) and *in vitro *(C)**. Bacterial colony forming units in BAL fluid (A) and blood (B) at 1, 3, and 7 days after injury in the E, F, and M groups. Data: mean ± SEM. (n = 10, 5, and 10 in the E, F, and M groups, respectively). (C) Bacterial colony forming units in media after 6 h incubation of *E. coli *with culture media only (E), fibroblasts (F), and human UCB-derived MSCs (M). Data: mean ± SEM. (n = 8, 8, and 8 in the E, F, and M groups, respectively).

To evaluate the bacterial burdens, the number of colony forming units (CFUs) was counted in BAL fluids and blood from animals in the E, F, and M groups at post-injury days 1, 3, and 7. Bacterial counts in both BAL and blood were significantly lower in M compared to E at post-injury day 7 (M, *p *< 0.01 vs. E); however, bacterial counts did not different between F and E at post-injury day 7 (Figure 8A-B).

Bacterial growth depending on the presence of MSCs was evaluated *in vitro*. Six hours after incubation, bacterial growth was significantly inhibited by MSC (M, *p *< 0.01 vs. E and F), but not by fibroblast (F, *p *> 0.05 vs. E) (Figure 8-C).

## Discussion

A major risk of ALI/ARDS is infection, such as severe sepsis or pneumonia, which causes most cases of mortality [2-4,21]. Lipopolysaccharide (LPS)-induced lung injury has been used as an animal model to mimic infectious human ALI and to stimulate host inflammatory responses [19,22-24]. However, LPS-induced ALI causes endothelial or epithelial injury that is less severe than that seen in human ALI [22]. In contrast, instillation of *E. coli *into mouse lungs resulted in ALI with a presentation similar to that of human ALI, including severe pneumonia and sepsis resulting in bilateral lung edema, alveolar hemorrhage, leukocyte infiltration, and alveolar wall thickening with severe inflammatory responses. Severe inflammatory responses were evidenced by lung MPO activity that was 20-fold greater than that of LPS-induced lung injury [25], a surge of inflammatory cytokines in lung homogenates, and increased water content in the lungs corresponding to an increased wet-dry lung ratio that peaked at post-injury day 3. Furthermore, the mortality rate in *E. coli*-induced ALI mice was about 30% with antibiotics treatment, which is consistent with infection related-ARDS mortality in clinical settings [4]. Overall, these findings suggest that *E. coli*-induced ALI resulting in severe inflammation is the most clinically relevant model to date.

In this study, intratracheal transplantation of human UCB-derived MSCs into *E. coli*-induced ALI mice significantly increased survival and attenuated lung injuries. The protective effect of MSCs was evidenced by decreased injury scores at post-injury day 3 and attenuated lung inflammation, including a reduction in MPO activity and inflammatory cytokine protein levels, at post-injury days 3 and 7. MSC transplantation also reduced the elevated lung water content at post-injury day 3 and bacterial counts in blood and BAL fluid at post-injury day 7 *in vivo *as well as *in vitro*. Thus, our results support the potential use of human UCB-derived MSCs as a new therapeutic modality for *E. coli*-induced ALI.

Although the mechanism underlying the therapeutic effect of MSCs on ALI has yet to be elucidated, the anti-inflammatory properties of MSCs may contribute to their protective role in ALI [7,8]. Recent ALI studies using BM-MSCs have also suggested their anti-inflammatory effects as the main protective mechanism [9,19,26,27]. Moreover, in previous studies, we successfully xenotransplanted human UCB-derived MSCs into immunocompetent newborn rats and demonstrated their efficacy in reducing hyperoxic lung injury, mainly through anti-inflammatory effects [14,15]. Here, we demonstrate attenuated lung MPO activity, which is an indicator of neutrophil accumulation or activity, resulting from intratracheal human UCB-derived MSC transplantation into ALI mice. The study also investigated inflammatory cytokine levels from lung homogenates as possible direct markers of lung inflammation. Protein levels of pro-inflammatory cytokines including IL-1α, IL-1β, IL-6, TNF-α, and MIP-2 were consistently reduced by MSC transplantation in ALI mice. These results clearly indicate an association between the attenuation caused by human UCB-derived MSCs in *E. coli*-induced ALI and these anti-inflammatory effects of these cells.

We used a protein macroarray to analyze lung inflammatory cytokines to elucidate the possible mechanism underlying the down-modulation of inflammation by MSCs in ALI mice. MSC transplantation showed suppressed pro-inflammatory molecules (IL-1α, IL-1β, IL-6, TNF-α), down-modulated the chemotactic effects of neutrophils, immature dendrocytes, and natural killer cells (MIP-1α, MIP-1β, RANTES), and decreased the chemotactic effects of T-cells (IP-10). These results indicate an overall decrease in Toll-like receptor (TLR) signaling [28-30]. TLRs are common immune molecules that recognize bacterial pathogens in acute lower respiratory bacterial infections [28]. Moreover, TLRs are pattern recognition receptors that control lung homeostasis and play a key role in both infectious and sterile lung inflammation [31]. Thus, human UCB-derived MSCs may modulate TLR signaling to attenuate the inflammation caused by *E. coli*-induced ALI. Another innate immune protein class that can either amplify or dampen TLR-induced signals is triggering receptor expressed on myeloid cells (TREM) [32]. TREM-1 is a well-known inflammatory amplifier [31]; thus, MSCs might also modulate the TREM-1 pathway that cross-reacts with TLR signaling. Other lung proteins attenuated by human UCB-derived MSCs, such as MCP-1 (JE), MCP-5, IL-16, and TIPM-1, participate in inflammatory processes by T cell activation and cell migration. Although the proteins presented in this study do not form a clear pathway network, down-regulation of *E. coli*-induced ALI by intratracheal transplantation of human UCB-derived MSCs was clearly associated with the control of complex inflammatory interactions.

Human UCB-derived MSCs seem to have an impact on endothelial and epithelial homeostasis in ALI. The increased wet-dry lung ratio in ALI implies lung edema which is primarily due to disrupted alveolar barrier integrity maintained by both the lung endothelium and epithelium. In this study, treatment with human UCB-derived MSCs improved the wet-dry lung ratio in ALI mice, suggesting that UCB-derived MSCs might have a role in repairing alveolar barrier integrity. The epithelial sodium channel (ENaC) and Na-K ATPase in type II pneumocytes have been postulated as candidate sites important for modulation by MSCs [33], probably by paracrine effects such as keratinocyte growth factor (KGF) [23]. Endothelial permeability modulation may be another mechanism of action of MSCs, and KGF and angiopoietin-1 are potentially important molecules involved in this effect [7,34-36]. Although MSCs have been shown to uniformly improve wet-dry lung ratios in ALI models [23,24,26], the precise action of MSCs on alveolar barrier integrity needs to be further investigated.

In this study, human UCB-derived MSC transplantation reduced bacterial concentration in bronchoalveolar spaces and blood of *E. coli*-induced ALI mice, and *in vitro *data also support an inhibiting effect of bacterial growth by human UCB-derived MSCs, not by fibroblasts, which is consistent with the study by Mei et al. in a sepsis model [9]. Recently, a study using BM-MSCs in a bacteria-induced ALI model suggested a paracrine effect by an antibacterial peptide from MSCs [10]. Because inflammatory cells contain many antibacterial peptides, MSCs might augment the antibacterial activity of those cells or secrete antibacterial peptides directly. Because little is known about the antibacterial activities of MSCs, additional *in vivo *and *in vitro *studies should be conducted to confirm the utility of MSCs in the treatment of bacterial diseases.

As a limitation of the study, we performed tissue examinations only in surviving animals at post-injury days 1, 3, and 7. Thus, this method may contribute to the lack of a difference at day 7, possibly resulting in an under-estimation of the therapeutic effect of MSCs.

Nevertheless, this study is unique in establishing the *E. coli *induced acute lung injury animal model and testing the protective anti-inflammatory and bactericidal effects of local intra-tracheal xeno-transplantation of human UCB derived MSCs *. E. coli *induced ALI animal model is essential for further studies to elucidate the mechanism of protective anti-inflammatory and bactericidal effects of human UCB derived MSCs observed in the present study in the near future.

## Conclusions

This study suggests that intratracheal transplantation of human UCB-derived MSCs into mice with *E. coli*-induced ALI significantly improves survival and attenuates ALI, primarily through anti-inflammatory mechanisms. Moreover, it is thought that human UCB-derived MSCs might have additional beneficial effects on *E. coli*-induced ALI, such as alveolar epithelial barrier repair and bacterial clearance. UCB is a clinically promising source of MSCs and our findings suggest human UCB-derived MSCs transplantation as a new therapeutic modality for reducing the high mortality and morbidity of human ALI.

## List of abbreviations

ALI: acute lung injury; ARDS: acute respiratory distress syndrome; BAL: bronchoalveolar lavage; *E. coli: Escherichia coli*; MPO: myeloperoxidase; MSC: mesenchymal stem cell; UCB: umbilical cord blood

## Competing interests

The authors declare that they have no competing interests.

## Authors' contributions

ESK: performed animal experiments, the statistical analyses, and wrote the manuscript.

YSC: supervised the study and participated in analyses and writing.

SJC: involved in study design and prepared MSCs.

JKK: performed animal experiments and the statistical analyses.

HSY: performed animal experiments and the statistical analyses.

SYA: performed animal experiments and the statistical analyses.

DKS: performed ELISA and macroarray analysis

SYK: performed animal experiments, histology and scoring, analyzed the results.

YRP: carried out animal experiments.

WSP: conceived, supervised and coordinated the study.

All authors contributed to the analyses and interpretation of the data. All authors have read and approved the final manuscript.
